# 小细胞肺癌目前治疗的策略与未来方向

**DOI:** 10.3779/j.issn.1009-3419.2017.06.09

**Published:** 2017-06-20

**Authors:** 为朋 邵, 晓伟 王, 德若 刘

**Affiliations:** 100029 北京，北京大学中日友好临床医学院胸外科 Department of Thoracic Surgery, China-Japan Friendship Hospital, Peking University Health Science Center (PUSHC), Beijing 100029, China

**Keywords:** 肺肿瘤, 手术, 化疗, 放疗, 靶向治疗, 免疫治疗, Lung neoplasms, Surgery, Chemotherapy, Radiotherapy, Target therapy, Immunotherapy

## Abstract

小细胞肺癌是一种致死率较高的恶性肿瘤，现阶段的治疗方式有手术，化疗和放疗，但预后极差。近些年来涌现的靶向治疗和免疫治疗也都在进行着大量的临床试验，本文将对小细胞肺癌目前的治疗策略以及未来研究的方向进行综述。

## Abstract (en)

Small cell lung cancer (SCLC) is a malignant tumor with a very high mortality rate. The current standard of care includes surgery, chemotherapy and radiotherapy. The clinical benefit of therapies was disappointing. Recently, many clinical trials about target therapy and immunotherapy are processing. This review will focus on current therapy and future research direction of SCLC.

肺癌是我国最常见的恶性肿瘤，全国肿瘤登记中心2016年发布的数据显示，肺癌在男性患者中占据第一位，女性患者也仅次于乳腺癌^[1]^。主要类型包括非小细胞肺癌（non-small cell lung cancer, NSCLC）和小细胞肺癌（small cell lung cancer, SCLC），SCLC占新发肺癌的10%-15%，是一种侵袭性较高的神经内分泌肿瘤，在临床和病理特点上明显不同于NSCLC，具有生长迅速、容易耐药、较早转移等特点，几乎所有的SCLC患者都有严重吸烟史^[2, 3]^。

## 1 SCLC目前主要治疗策略

SCLC目前的治疗方式主要有手术、化疗、放疗和尚未正式批准运用到临床的免疫及靶向治疗。由于SCLC的临床特点，发现时已有远处转移，仅有约5%的SCLC患者能够早期发现并能完成手术治疗，而且手术治疗仅适用于临床分期I期（T1-2, N0），并且纵隔淋巴结未被侵犯的局限期SCLC患者^[4]^。对于手术方式的选择肺叶切除优于肺段或楔形切除术（*P*=0.03）^[5]^，肿瘤切除后推荐辅助化疗或者放化疗联合治疗^[6]^。化疗是各种治疗方式的基本组成部分，目前对于局限期一线化疗方案主要为顺铂+依托泊苷（EP），广泛期的化疗方案主要有铂类+伊立替康和EP方案，来自日本的Ⅲ期临床试验发现对于广泛期SCLC铂类+伊立替康比EP方案的中位生存期更长（12.8 months *vs* 9.4 months, *P*=0.02）^[7]^，然而与之相反另外一项Ⅲ期临床试验2种治疗方案并没有显著的临床差异^[8]^。虽然SCLC对化疗敏感，但化疗后容易产生耐药和复发，复发时间间隔大于6个月的继续应用原方案，间隔小于6个月的根据化疗后复发的情况二线化疗方案主要有拓扑替康、伊立替康、紫杉醇、多西紫杉醇、口服依托泊苷、吉西他滨、及诺维本等药物^[9]^。胸部放疗可以提高局限期SCLC患者的生存率，放化疗联合治疗相比化疗单药治疗更能使患者获益，但传统的放化疗对于局限期SCLC患者的长期生存仍是巨大的挑战，对于广泛期SCLC患者，有研究表明若患者对化疗有反应，可以顺序施加放疗，一项Ⅲ期临床试验发现化疗后再放疗相比没有接受放疗的患者并没有提高1年的总生存率（33% *vs* 28%, *P*=0.066），但提高了2年的总生存率（13% *vs* 3%, *P*=0.004）^[10]^。超过50%的SCLC患者会发生脑转移，预防性脑照射（prophylactic cranial irradiation, PCI）可以阻止脑转移的发生，治疗中发挥着重要作用，一项回顾性临床研究发现PCI对比没有PCI的局限期SCLC患者更能提高其生存率，同步放化疗是治疗SCLC的标准方案，放疗应尽早应用到SCLC的治疗中，在1个-2个化疗周期后即可开始放疗，缩短任何治疗方式同放射治疗的间隔，均可明显提高患者的生存率^[11]^，对于广泛期SCLC患者，结果令人失望，一项来自日本的Ⅲ期临床试验PCI后并不能提高其生存率^[12]^。关于肿瘤标记物相关临床试验发现核小体，胃泌素释放肽（gastrin releasing peptide, ProGRP）、神经元特异性烯醇化酶（neuron specific enolase, NSE）、细胞角蛋白19片段（cytokeratin 19 fragment, CYFRA21-1）肿瘤标记物在初始治疗过程保持在较高的水平或者下降不明显会有较差的治疗结果，这些肿瘤标记物或许可以在治疗周期中作为疗效的监测方式^[13]^。放化疗及手术均为SCLC的主要治疗方式，但SCLC的总体生存率仍较低，现有的治疗方式已达到瓶颈，但随着生物免疫靶向治疗方式的出现，SCLC的患者或许能见到一丝曙光。

## 2 SCLC靶向治疗研究进展

靶向治疗的原理是通过在基因分子层面改变肿瘤的信号传导途径、影响血管生成等各种方式从而抑制肿瘤细胞的生长。靶向药物[如针对表皮生长因子受体（epidermal growth factor receptor, *EGFR*）突变和间变性淋巴瘤激酶（anaplastic lymphoma kinase, *ALK*）基因重排]在NSCLC中已经得到广泛应用，可以显著改善患者的预后及生存质量。SCLC基因组中表现了较高的基因突变率，如TP53、RB1、NOCTH家族的失活性突变，*TP73*的基因突变和基因组重排，*EGFR*、*KRAS*的激活性突变，*MYC*扩增^[14, 15]^。相关研究完成了大量的临床试验，但仍没有明确针对此肺癌亚型的靶向药物。在本节中，我们重点报道在基因层面的临床试验和有针对性的方法，以研究SCLC的靶向治疗的未来方向。

### 2.1 酪氨酸激酶受体（receptor tyrosine kinases, RTKs）

RTKs是细胞信号传递过程的关键调控因子，在细胞增殖和分化、细胞的生存和代谢、器官形态、新生血管形成、细胞迁移、组织修复和再生定义中起着重要作用^[16]^。异常RTKs信号是肿瘤起始和进展的重要特征，所以RTKs可以作为未来研究SCLC的重要分子靶点。

#### 2.1.1 c-KIT抑制剂

c-KIT抑制剂如甲磺酸伊马替尼（imatinib mesylate）通过占领酪氨酸激酶与ATP结合位点从而抑制酪氨酸激酶活性，伊马替尼活性与9号和11号外显子突变相关，此类基因突变在胃肠间质瘤中比较常见^[17]^。在一项Ⅱ期临床试验中，入组19例患者，分别将伊马替尼用到广泛期SCLC和化疗后复发的SCLC 2组患者中，伊马替尼不能有效控制疾病进展，并且只有21%肿瘤样本中表达c-kit^[18]^。在另外一项Ⅱ期临床试验中，收集29例入组标准为c-kit阳性的SCLC患者，c-kit抑制剂同样未能证实其临床效果^[19]^。

#### 2.1.2 EGFR抑制剂（EGFR tyrosine kinase inhibitors, EGFR-TKIs）

EGFR在许多肿瘤细胞中过度表达，在NSCLC中靶向药物已经运用到临床，而在SCLC中EGFR则表达较少甚至不表达^[20]^。在一项Ⅱ期临床试验中，纳入20例SCLC患者，接受EGFR-TKIs吉非替尼治疗后，无患者获得完全缓解（complete response, CR）或者部分缓解（partial response, PR），2例患者获得疾病稳定（stable disease, SD），其余患者均发生不同程度的疾病进展（progressive disease, PD），此项研究并未证明EGFR-TKIs能够抑制肿瘤的生长，作者认为SCLC的生长或许并不依赖细胞增殖周期中的EGFR的表达^[21]^。

#### 2.1.3 胰岛素样生长因子[insulin-like growth factor-1（IGF1）/IGF-1 receptor（IGF-1R）]

IGF1/IGF-1R是一种恶性肿瘤表达的启动子，通过PI3K/Akt和MAPK通路传递信号，通过抑制PI3K/AKT和MAPK通路可以增强化疗的效果。一项临床研究证明IGF1/IGF-1R抑制剂同步联合铂类化疗药以及放疗可以明显提高患者总生存率^[22]^。IGF1/IGF-1R抑制剂在动物模型实验中可使肿瘤获得明显的缩小，或许此类药可以进行下一步的临床试验^[23]^。

#### 2.1.4 成纤维细胞生长因子受体（fibroblast growth factor receptor, FGFR）

FGFR通过受体间相互作用来介导胚胎的发育，在组织的修复以及系统的动态平衡中起着重要作用。在乳腺癌、食管癌以及头颈部癌较常见编码*FGFR*的基因改变（如基因扩增、突变、移位和表达增加）^[24]^，Schultheis等^[25]^研究发现大约有5%的SCLC患者中FGFR-1高表达，应该积极推进关于FGFR抑制剂的临床试验研究^[26]^。在体内外试验中应用FGFR抑制剂可以抑制SCLC细胞的生长，或许能为SCLC提供新的思路。

#### 2.1.5 MET抑制剂

MET也是一种RTKs，在SCLC中过度表达，通过HGF/MET轴发挥作用，对正常细胞和恶性细胞的信号传递均发挥重要作用。一项对照试验表明，MET抑制剂联合拓扑异构酶抑制剂均比两项单药治疗能够明显缩小肿瘤的大小（*P* < 0.05），为SCLC患者的治疗提供了潜在的有效的措施^[27]^。

### 2.2 靶向Hedgehog（Hh）通路

Hh通路分泌的蛋白质参与调节细胞的生长、分化和增殖，然而如果通路由于异常的基因突变或者其他机制导致其在成人器官或者组织中过度活跃，对于各个器官肿瘤的发生尤其是肺组织起了重要作用^[28]^。Park等^[29]^利用小鼠模型，通过敲除小鼠的*Rb1*和*Trp53*基因诱导SCLC的发生，发现Hh通路是影响SCLC细胞微环境的独立因素，药物阻断Hh通路可以抑制SCLC的生长，为SCLC的靶向药物治疗提供了新的方向，而且很多Hh通路抑制剂正处于临床试验中。

### 2.3 PI3K/AKT/mTOR通路

PI3K/AKT/mTOR通路参与细胞增殖、分化、凋亡和葡萄糖转运等多种细胞功能的调节，通路活性的增加常与多种癌症的发生相关。一项来自日本的回顾性研究筛选了从1992年-2012年间1, 042例SCLC患者，共有55例SCLC入组，研究发现碱基G与碱基T间的替换是主要的突变类型，大约有40%的SCLC患者有PI3K/AKT/mTOR通路的异常，并且在体外应用此通路抑制剂可以明显减弱SCLC的增殖^[30]^。PI3K/AKT/mTOR通路抑制剂如everolimus和temsirolimus都正在处于Ⅱ期临床试验中，并且everolimus已经运用到肾细胞癌的靶向治疗中^[31, 32]^。

### 2.4 血管生成通路

血管的生成对肿瘤的生长以及远处播散起到了关键作用，血管内皮生长因子（vascular endothelial growth factor, VEGF）是血管在正常生理情况和异常病理情况的关键参与者。贝伐珠单抗（Bevacizumab, Bev）是重组人源化抗VEGF的单克隆抗体，已被批准用于一线治疗几种实体肿瘤，例如NSCLC、乳腺癌、结肠癌、肾细胞癌和卵巢癌。Trafalis等^[33]^Ⅱ期临床试验，以顺铂+依托泊苷治疗后复发的病例，采用伊立替康+贝伐珠单抗二线治疗，总体反应率（overall response rate, ORR）包括PR和CR为25%（95%CI: 8.9-41.0），SD患者2个月总疾病控制率为89%（95%CI: 77.41-100），中位反应时间为6个月，中位数无进展生存期为3个月（mean PFS: 3.2 months, 95%CI: 2.7-3.7），6个月的无进展生存率3.6%，1年OS率为3.6%，认为化疗后复发的SCLC患者合用贝伐珠单抗是一种有效的治疗措施，但进一步的大规模的研究仍是有必要的。一项更大数量的多中心、Ⅲ期、随机对照临床试验研究对比顺铂+依托泊苷与顺铂+依托泊苷+贝伐珠单抗作为一线治疗广泛期SCLC，204例SCLC患者入组，随机分为2组，中位随访期为34.9个月，2组中位OS分别为8.9个月和9.8个月，1年生存率为25%和37%（HR=0.78; 95%CI: 0.58-1.06; *P*=0.113），接受贝伐珠单抗的患者的OS有显著的统计学差异（HR=0.60; 95%CI: 0.40-0.91; *P*=0.011），中位PFS分别为5.7个月和6.7个月（*P*=0.030），在一线治疗中加入贝伐珠单抗组PFS有获益，OS只在少数维持治疗的患者中获益^[34]^。贝伐珠单抗从试验到临床应用仍需大量的临床研究来证实其安全性、有效性。

### 2.5 Aurora激酶抑制剂

Aurora激酶是负责调控细胞有丝分裂的一类重要的丝氨酸/苏氨酸激酶，异常表达（比如*Rb*基因的失活，MYC家族的扩增）的Aurora激酶往往会导致细胞在有丝分裂的过程中出现大量的异常现象，Barbara等在体内外试验发现SCLC基因中MYC的扩增或者高度表达将会对Barasertib（Aurora激酶抑制剂）有较高的反应，MYC的表达产物可作为对SCLC的疗效预测标记物^[35]^。Mollaoglu等^[36]^通过使小鼠MYC基因表达同时*Rb1*和*Trp53*基因的缺失造成小鼠多发转移、侵袭性较强状态的肿瘤，并用类似人类SCLC的化疗药物进行治疗，之后联合Aurora激酶抑制剂可以明显压制肿瘤的进展和提高生存率。

### 2.6 PARP抑制剂

PARP是一种多功能蛋白翻译后修饰酶，在DNA损伤修复和细胞凋亡中发挥着重要作用，通过识别结构损伤的DNA片段而被激活，被视为DNA损伤的感受器^[37]^。体外研究发现在SCLC中PARP的水平会上调，上调的水平与其对PARP抑制剂的敏感性相关^[38]^。Lok等^[39]^发现在体内试验中SLFN11可以作为PARP抑制剂的疗效评价指标，替莫唑胺已被推荐SCLC复发转移的治疗方案，PARP抑制剂联合替莫唑胺可以使SCLC增加控制的程度，并且可以减少单药治疗的不良反应。但是未来的临床研究仍需要证实这项临床前期研究。Stewart等^[40]^通过体外试验发现在应用卡铂联合PARP抑制剂时，可以使体外试验获得明显获益并且可以通过SLFN11、EMT和ATM三种疗效预测标记物的变化来预测其效果。

### 2.7 抗偶联药物Rova-T

抗偶联药物Rova-T是针对DLL-3（Delta-like ligand 3）蛋白表达的靶向制剂，DLL-3蛋白参与肿瘤干细胞的调节，高度表达在神经内分泌肿瘤的干细胞表面。Rovalpituzumabtesirine（Rova-T）是抗DLL3蛋白抗体Rovalpituzumab与细胞毒素Tesirine的偶联药物。Saunders等^[41]^已经完成了体内的1期临床研究，Rova-T对SCLC具有一定的抗肿瘤活性，为下一步2期研究奠定了基础，并且DLL-3蛋白可以作为SCLC的疗效预测标志物。

## 3 SCLC免疫治疗研究进展

目前针对SCLC患者的治疗仍然是沿用30年来一致的以放化疗为主的治疗，虽然最近靶向治疗的药物越来越多的进行了临床试验，但距离运用到临床仍然需要一定的时间，这就迫使临床医生研究新的治疗方式和方法，随着分子生物学、免疫学及基因工程技术在肿瘤学中的研究深入和应用，肿瘤免疫治疗已经成为肿瘤治疗领域的研究热点。近年来在肺癌领域很多免疫治疗也随之发展，到目前为止，免疫检查点抑制剂（immune checkpoint inhibitors）通过抑制肿瘤细胞逃避免疫监视和识别的作用，成为免疫治疗最有前景的方式。

### 3.1 程序性死亡受体-1（programmed cell death protein-1, PD-1）

Schultheis等^[42]^研究94例SCLC患者发现肿瘤细胞的PD-1受体均为阴性，而肿瘤的间质细胞（肿瘤浸润的巨噬细胞和淋巴细胞）则发现了受体的表达，抗PD-1抗体或许对肿瘤间质表达PD-1的SCLC发挥作用，并且作者认为肿瘤微环境的研究应该被纳入临床试验。但Komiya对上述研究提出了反对意见，通过对99例SCLC患者分析，得出有82例患者表达阳性结果即有PD-1表达，如此高的阳性率得到了Ishii等研究的支持，Ishii等通过免疫组化分析102例SCLC患者，其中73例（71.6%）表达PD-1，SCLC患者PD-1阳性的明显比阴性的有更好的OS，与PFS没有直接的关系^[43, 44]^。而Chang等^[45]^研究PD-1在肿瘤细胞和肿瘤浸润的淋巴细胞分别为78.0%和54.3%，与Ishii相反的是PD-1阳性的SCLC患者中却为更差的OS。

### 3.2 细胞毒性T淋巴细胞抗原-4（cytotoxic T lymphocyte antigen 4, CTLA-4）

CTLA-4作用机制和PD-1类似，通过参与免疫反应的负调节来发挥作用。伊匹单抗（ipilimumab）是一种抗CTLA-4的单克隆抗体，已被美国食品和药物管理局（Food and Drug Administration, FDA）批准运用到恶性黑色素瘤的免疫治疗。SCLC的相关研究也逐渐进行，Reck等^[46]^纳入334例SCLC患者行Ⅱ期临床试验认为伊匹单抗联合紫杉醇+卡铂对比单用紫杉醇+卡铂可以延长OS（12.9个月*vs* 9.9个月；HR=0.75；*P*=0.13），同时可以提高免疫相关PFS（immune-related PFS, irPFS）（HR=0.64; *P*=0.03）。随后Reck等又做了一项1, 132例SCLC患者的Ⅲ期临床试验，化疗药物+伊匹单抗与化疗药物+安慰剂的中位OS分别为11.0个月和10.9个月（HR=0.94; 95%CI: 0.81-1.09; *P*=0.377, 5），中位PFS分别为4.6个月和4.4个月（HR=0.85; 95%CI: 0.75-0.97），化疗过程中加入伊匹单抗并不能提高患者OS，不过仍有大量的关于CTLA-4临床试验正在进行中：一线化疗后SCLC的进一步研究（NCT02538666）；抗PD-1抗体（Nivolumab）和抗CTLA-4抗体（Ipilimumab）联合在LD-SCLC中临床试验（NCT02046733）^[47]^。

单一方式治疗SCLC较为困难并且容易耐药，患者要承受单药大剂量带来的耐药性及严重不良反应，所以各种方式的联合治疗已有相关研究发现这种方式的优点。Konstantinos等^[48]^进行的化疗后加入免疫治疗药物的Ⅱ期随机对照研究，纳入164例患者，试验发现仅有局限期SCLC患者应用IFN-α组出现*P* < 0.05。Antonia等^[49]^发表了一项多中心、开放的I期/Ⅱ期临床试验，216例患者纳入试验，分成Nivolumab单药和nivolumab+ipilimumab联合，单药组有10%的反应率、32%的疾病控制率、1年生存率33%，联合组有19%-23%的反应率、36%-42%的疾病控制率、1年生存率35%-43%，这些数据表明在有限的治疗措施中2种免疫药物联合不失为一种可供选择的治疗^[50]^。相比其他肿瘤，SCLC的PD-L1阳性率极低，或许2药联合中ipilimumab激活了nivolumab的活性。但在另外一项Ⅲ期临床试验中，将SCLC患者分成ipilimumab单药和ipilimumab联合化疗药物，对比各项发现2组并没有明显的临床疗效差别，这就意味着ipilimumab在SCLC中发挥着有限的作用。

## 4 SCLC的其他药物

阿司匹林作为一种老药新用，已被证实可以通过抑制环氧合酶和减少前列腺素的合成来减少结直肠癌的发病率和死亡率^[50]^。长期观察发现阿司匹林的应用在其他肿瘤包括肺癌同样可以减少死亡率，所以一项关于SCLC的前瞻性队列研究系统地研究了阿司匹林的作用，作者发现长期低剂量的服用阿司匹林并不能提高SCLC患者的生存率，或许与SCLC中环氧合酶-2低水平有关^[51]^。

## 5 结语

目前针对SCLC的治疗仍然是延续近30年传统的放化疗为基础的治疗，鉴于SCLC的异质性以及基因组的复杂性，首先要从诊断入手提高SCLC的诊出率，比如联合传统的细胞学、免疫组化、二代测序甚至三代测序等方式。其次针对当下的靶向药物的临床试验并不能明显改善患者的预后以及免疫治疗的蓬勃发展，多方式综合治疗即放化疗+靶向治疗+免疫治疗或许可以给SCLC患者带来一线生机。最后，SCLC在分子病理的机制仍未完全明确，仍需技术的进步给予分子层面的解释，仍需大量的临床试验进行新药的研发，仍需新的治疗策略指导临床实践。

## References

[1] Chen W, Zheng R, Baade PD (2016). Cancer statistics in China, 2015. CA Cancer J Clin.

[2] Siegel RL, Miller KD, Jemal A (2017). Cancer statistics, 2017. CA Cancer J Clin.

[3] Pesch B, Kendzia B, Gustavsson P (2012). Cigarette smoking and lung cancer-relative risk estimates for the major histological types from a pooled analysis of case-control studies. Int J Cancer.

[4] Schneider BJ, Saxena A, Downey RJ (2011). Surgery for early-stage small cell lung cancer. J Natl Compr Canc Netw.

[5] Brock MV, Hooker CM, Syphard JE (2005). Surgical resection of limited disease small cell lung cancer in the new era of platinum chemotherapy: Its time has come. J Thorac Cardiovasc Surg.

[6] Yang CF, Chan DY, Speicher PJ (2016). Role of adjuvant therapy in a population-based cohort of patients with early-stage small-cell lung cancer. J Clin Oncol.

[7] Noda K, Nishiwaki Y, Kawahara M (2002). Irinotecan plus cisplatin compared with etoposide plus cisplatin for extensive small-cell lung cancer. N Engl J Med.

[8] Lara PN, Jr., Natale R, Crowley J (2009). Phase Ⅲ trial of irinotecan/cisplatin compared with etoposide/cisplatin in extensive-stage small-cell lung cancer: clinical and pharmacogenomic results from SWOG S0124. J Clin Oncol.

[9] Owonikoko TK, Behera M, Chen Z (2012). A systematic analysis of efficacy of second-line chemotherapy in sensitive and refractory small cell lung cancer. J Thorac Oncol.

[10] Slotman BJ, van Tinteren H, Praag JO (2015). Use of thoracic radiotherapy for extensive stage small-cell lung cancer: a phase 3 randomised controlled trial. Lancet.

[11] Patel S, Macdonald OK, Suntharalingam M (2009). Evaluation of the use of prophylactic cranial irradiation in small cell lung cancer. Cancer.

[12] Seto T, Takahashi T, Yamanaka T (2014). Prophylactic cranial irradiation (PCI) has a detrimental effect on the overall survival (OS) of patients (pts) with extensive disease small cell lung cancer (ED-SCLC): results of a Japanese randomized phase Ⅲ trial. J Clin Oncol.

[13] Holdenrieder S, Pawel JC, Dankelmann E (2008). Nucleosomes, ProGRP, NSE, CYFRA 21-1, and CEA in monitoring first-line chemotherapy of small cell lung cancer. Clin Cancer Res.

[14] George J, Lim JS, Jang SJ (2015). Comprehensive genomic profiles of small cell lung cancer. Nature.

[15] Semenova EA, Nagel R, Berns A (2015). Origins, genetic landscape, and emerging therapies of small cell lung cancer. Genes Dev.

[16] Ullrich A, Schlessinger J (1990). Signal transduction by receptors with tyrosine kinase activity. Cell.

[17] Blay JY, Casali PG, Dei Tos AP (2015). Management of gastrointestinal stromal tumour: current practices and visions for the future. Oncology.

[18] Johnson BE, Fischer T, Fischer B (2003). Phase Ⅱ study of imatinib in patients with small cell lung cancer. Clin Cancer Res.

[19] Dy GK, Miller AA, Mandrekar SJ (2005). A phase Ⅱ trial of imatinib (ST1571) in patients with c-kit expressing relapsed small-cell lung cancer: a CALGB and NCCTG study. Ann Oncol.

[20] Haeder M, Rotsch M, Bepler G (1988). Epidermal growth factor receptor expression in human lung cancer cell lines. Cancer Res.

[21] Moore AM, Einhorn LH, Estes D (2006). Gefitinib in patients with chemo-sensitive and chemo refractory relapsed small cell cancers: A Hoosier Oncology Group phase Ⅱ trial. Lung Cancer.

[22] Ferté C, Loriot Y, Clémenson C (2013). IGF-1R targeting increases the antitumor effects of DNA-damaging agents in SCLC model: an opportunity to increase the efficacy of standard therapy. Mol Cancer Ther.

[23] Zinn RL, Gardner EE, Marchionni L (2013). ERK phosphorylation is predictive of resistance to IGF-1R inhibition in small cell lung cancer. Mol Cancer Ther.

[24] Brooks AN, Kilgour E, Smith PD (2012). Molecular pathways: fibroblast growth factor signaling: a new therapeutic opportunity in cancer. Clin Cancer Res.

[25] Schultheis AM, Bos M, Schmitz K (2014). Fibroblast growth factor receptor 1 (FGFR1) amplification is a potential therapeutic target in small-cell lung cancer. Mod Pathol.

[26] Pardo OE, John Latigo, Jeffery RE (2009). The fibroblast growth factor receptor inhibitor PD173074 blocks small cell lung cancer growth *in vitro* and *in vivo*. Cancer Res.

[27] Rolle CE, Kanteti R, Surati M (2014). Combination MET inhibition and Topoisomerase I inhibitionblock cell growth of Small Cell Lung Cancer. Mol Cancer Ther.

[28] Huang S, Yang L, An Y (2011). Expression of hedgehog signaling molecules in lung cancer. Acta Histochem.

[29] Park KS, Martelotto LG, Pefer M (2011). A crucial requirement for Hedgehog signaling in small cell lung cancer. Nat Med.

[30] Umemura S, Mimaki S, Makinoshima H (2014). Therapeutic priority of the PI3K/AKT/mTOR pathway in small cell lung cancers as revealed by a comprehensive genomic analysis. J Thorac Oncol.

[31] Tarhini A, Kotsakis A, Gooding W (2010). Phase Ⅱ study of everolimus (RAD001) in previously treated small cell lung cancer. Clin Cancer Res.

[32] Pandya KJ, Dahlberg S, Hidalgo M (2007). A randomised, phase Ⅱ trial of two dose levels of temsirolimus (CCI-779) in patients with extensive stage small-cell lung cancer who have responding or stable disease after induction chemotherapy: a trial of the Eastern Cooperative Oncology Group (E1500). J Thorac Oncol.

[33] Trafalis DT, Alifieris C, Stathopoulos GP (2016). Phase Ⅱ study of bevacizumab plus irinotecan on the treatment of relapsed resistant small cell lung cancer. Cancer Chemother Pharmacol.

[34] Tiseo M, Boni L, Ambrosio F (2017). Italian, multicenter, phase Ⅲ, randomized study of cisplatin plus etoposide with or without Bevacizumab as first-line treatment in extensive-disease small-cell lung cancer: The GOIRC-AIFA FARM6PMFJM Trial. J Clin Oncol.

[35] Helfrich BA, Kim J, Gao DX (2016). Barasertib (AZD1152), a small molecule Aurora B inhibitor, inhibits the growth of SCLC cell lines *in vitro* and *in vivo*. Mol Cancer Ther.

[36] Mollaoglu G, Guthrie MR, Bohm S (2016). MYC drives progression of small cell lung cancer to a variant neuroendocrine subtype with vulnerability to Aurora kinase inhibition. Cancer Cell.

[37] Rouleau M, Patel A, Hendzel MJ (2010). PARP inhibition: PARP1 and beyond. Nat Rev Cancer.

[38] Averett BL, Wang J, Monique BN (2012). Proteomic profiling identifies dysregulated pathways in small cell lung cancer and novel therapeutic targets including PARP1. Cancer Discov.

[39] Lok BH, Gardner EE, Schneeberger VE (2017). PARP inhibitor activity correlates with SLFN11 expression and demonstrates synergy with Temozolomide in small cell lung cancer. Clin Cancer Res.

[40] Stewart CA, Tong P, Cardnell RJ (2017). Dynamic variations in epithelial-to-mesenchymal transition (EMT), ATM, and SLFN11 govern response to PARP inhibitors and cisplatin in small cell lung cancer. Oncotarget.

[41] Saunders LR, Bankovich AJ, Anderson WC (2015). A DLL3-targeted antibody-drug conjugate eradicates high-grade pulmonary neuroendocrine tumor-initiating cells *in vivo*. Sci Transl Med.

[42] Schultheis AM, Scheel AH, Ozretić L (2015). PD-L1 expression in small cell neuroendocrine carcinomas. Eur J Cancer.

[43] Komiya T, Madan R (2015). PD-L1 expression in small cell lung cancer. Eur J Cancer.

[44] IshⅡ H, Azuma K, Kawahara A (2015). Significance of programmed cell death-ligand 1 expression and its association with survival in patients with small cell lung cancer. J Thorac Oncol.

[45] Chang YL, Yang CY, Huang YL (2017). High PD-L1 expression is associated with stage Ⅳ disease and poorer overall survival in 186 cases of small cell lung cancers. Oncotarget.

[46] Reck M, Bondarenko I, Luft A (2013). Ipilimumab in combination with paclitaxel and carboplatin as first-line therapy in extensive-disease-small-cell lung cancer: results from a randomized, double-blind, multicenter phase 2 trial. Ann Oncol.

[47] Reck M, Luft A, Szczesna A (2016). Phase Ⅲ randomized trial of ipilimumab plus etoposide and platinum versus placebo plus etoposide and platinum in extensive-stage small cell lung cancer. J Clin Oncol.

[48] Konstantinos Z, Eftimios Z, Efimia B (2013). Immunomodifiers in combination with conventional chemotherapy in small cell lung cancer: a phase Ⅱ, randomized study. Drug Des Devel Ther.

[49] Antonia SJ, Lopez MJA, Bendell J (2016). Nivolumab alone and nivolumab plus ipilimumab in recurrent small-cell lung cancer (CheckMate 032): a multicentre, open-label. Lancet Oncol.

[50] Chan AT, Ogino S, Fuchs CS (2007). Aspirin and risk of colorectal cancer in relation to expression of COX-2. N Engl J Med.

[51] Maddison P (2017). Effects of aspirin on small-cell lung cancer mortality and metastatic. Lung Cancer.

